# Repellent properties of *Rotheca glabrum* plant extracts against adults of *Rhipicephalus appendiculatus*

**DOI:** 10.1186/s12917-019-1853-5

**Published:** 2019-04-27

**Authors:** Kedibone Gloria Mawela, Dibungi Luseba, Solomon Magano, Jacobus Nicolaas Eloff

**Affiliations:** 1 Phytomedicine Programme, Department of Paraclinical Sciences, University of Pretoria, Private Bag X04, Onderstepoort, 0110 Republic of South Africa; 2 Department of Animal Sciences, Faculty of Science, Tshwane University of Technology, Private Bag X680, Pretoria, 0001 South Africa; 3 Department of Life and Consumer Science, University of South Africa, Private Bag X6, Florida, 1710 Republic of South Africa; 4 Department Agriculture and Animal Health, University of South Africa, Private Bag X6, Florida, 1710 Republic of South Africa; 5 Agricultural Research Council, Onderstepoort Veterinary Research, Division of Toxicology, Private Bag X05, Onderstepoort, 0110 South Africa

**Keywords:** *Rotheca glabrum*, Infusion, Decoction, Repellent, Bioassay-guided fractionation

## Abstract

**Background:**

*Rotheca glabrum* (formerly known as *Clerodendrum glabrum* [Verbenaceae]) is used by local communities in the Limpopo Province of South Africa to control ticks on livestock and was selected from the database of the ARC-Onderstepoort Veterinary Institute. Its leaves were extracted using organic solvents ranging from polar to non-polar solvents (methanol, acetone and dichloromethane (DCM)). In addition, the traditional soap-water (infusion) and water-based (decoction) methods were used. The tick repelling activity was determined against the adult stage of the livestock tick *Rhipicephalus appendiculatus*.

**Results:**

In the tick-climbing repellency bioassay a 30% acetone extract had a significant (*p* ≤ 0.05) repellent effect against adults of *R. appendiculatus*. The extract was still active at a lower concentration of 10%. The hexane fraction from the *R. glabrum* acetone extract had a higher tick repellency activity than the positive controls Amitix and Bayticol at the same concentrations. Unfortunately, the activity decreased after 2.5 h, probably due to volatility of the biologically active compound(s) within the extract.

**Conclusion:**

Attempts were made to isolate the repellent compound from the acetone extract of *R. glabrum.* The process produced very good results up to a late stage in the bioassay-guided fractionation process. At that point, the repellent activity was lost. When two fractions were combined, the repellent activity was regained. These results provide strong evidence for the existence of a synergisticactivity of different compounds. It may be better to concentrate on extracts that would kill ticks rather than on extracts that would repel ticks.

## Background

Ticks belong to the phylum Arthropoda and are obligate hematophagus ecto-parasites of reptiles, birds and mammals throughout the world [1, 2]. They are divided taxonomically into three main families namely Argasidae (soft ticks), Nuttallielidae and Ixodidae (hard ticks) [3]. Tick bites continue to cause serious public health and management problems, especially in developing countries as they transmit viral, rickettsial, bacterial and protozoal disease-causing agents affecting wildlife, domestic animals and humans [4–6]. In particular, *R. appendiculatus* is a principal vector of *Theileria parva,* the causative agent of East Coast Fever (ECF), a non-contagious febrile lymphoproliferative disease of cattle [7, 8]. Although ECF was eradicated in South Africa by 1954, it still exists in numerous countries in Central, East and North Africa [9] causing high mortality and morbidity rates among cattle [10]. *R. appendiculatus* also transmits *T. taurotragi*, *Anaplasma marginale*, Thogoto virus and *Rickettsia conorii* the causative agents of benign bovine theileriosis, bovine anaplasmosis, Nairobi sheep disease and tick typhus in humans, respectively [11].

Generally, it is agreed that ticks and tick-borne diseases pose a major threat to livestock industries throughout the world. Thus, ticks and tick-borne diseases in cattle have been estimated to cost the global industry between US$ 13.9 million and US$ 18.7 billion, annually [12]. *T. parva* has been implicated as a cause of production losses in excess of US$ 200 million per year in small scale and traditional farming communities of Kenya and Tanzania [13, 14]. While in 1993, ECF was estimated to cost Africa US$ 168 million [15]. Hence, there is an urgent need to find effective strategies to control tick infestations. Vaccines offer an alternative approach to control ticks and prevent tick-borne diseases as they have been shown to be efficient, cost-effective and environmentally friendly [16]. Currently, a live attenuated Muguga cocktail vaccine has been used in other African countries to protect cattle against ECF [17]. However, use of the vaccine results in a *T. parva* carrier-state in vaccinated cattle that may render them as a source of infective parasite to other livestock. Even though the vaccine provides a broad-spectrum immunity against ECF in cattle [18, 19], a further investigation of the effectiveness of the vaccine against buffalo-derived *T. parva* failed, hence the cattle died due to Corridor disease [20]. Moreover, the production of the vaccine requires highly complex, costly processes, including a liquid nitrogen cold chain [21].

The limitations associated with the current vaccine makes it unsuitable for use in South Africa. Therefore, application of chemical acaricides has been another component of integrated tick control. However, the use of the current suite of chemical acaricides is unsustainable, even in the medium term, as it is accompanied by serious drawbacks including selection of acaricide-resistant tick populations, environmental concerns and contamination of milk and meat products with drug residues [22, 23]. Therefore, there is considerable interest in developing natural plant-based repellents with greater efficacy and extended activity. *Rotheca glabrum* leaves have been used for their tick- and insect-repellent properties. Thus, local communities have crushed and mixed the leaves of the plant with water to prepare an infusion extract to administer per os [24, 25], while the decoction extract was prepared by boiling the mixture of the leaves and water for topical administration [24, 26]. However, the bioactive chemical components of the leaf extracts remain unexplored. Hence, the present study was conducted to investigate the repellency activity of *R. glabrum* extracts against adults of *R. appendiculatus.*

## Results

### General results

**Fig. 1:**
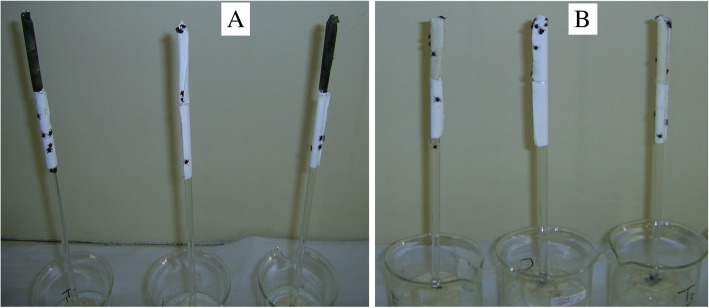
Example of a repellent (**a**) and a non-repellent (**b**) extract. Treatment bioassays, the top stained filter papers were coated with *R. glabrum* extract. All the bottom filter papers contained no extract or solvent. Control bioassays, unstained filter papers at the top were treated with the negative control (solvent). Rods containing repellent acetone extract resulted in *R. appendiculatus* adults being repelled from the treated filter papers to the neutral filter papers, whereas no repellency was observed in the control bioassays (**a**). The soap-water infusion of *R. glabrum* and solvent control did not repel the ticks from climbing up the rods to the top filter paper (**b**)

The acetone extract repelled the ticks, but the soap-water infusion of *R. glabrum* leaves did not repel the ticks (Fig. 1a and b).

**Fig. 2:**
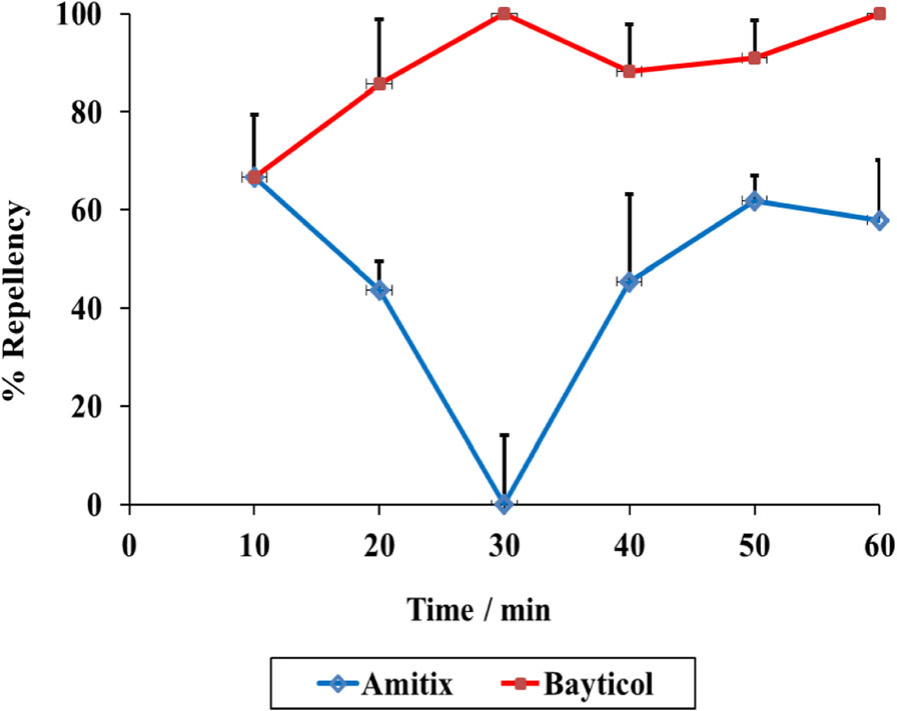
Percentage repellencies of the *R. appendiculatus* adults against 30% concentration of the positive controls as calculated from the adopted formula [44]

Bayticol and Amitix positive controls on adults of *R. appendiculatus* led to an average of 89 and 45% repellency, respectively (Fig. 2).

**Fig. 3:**
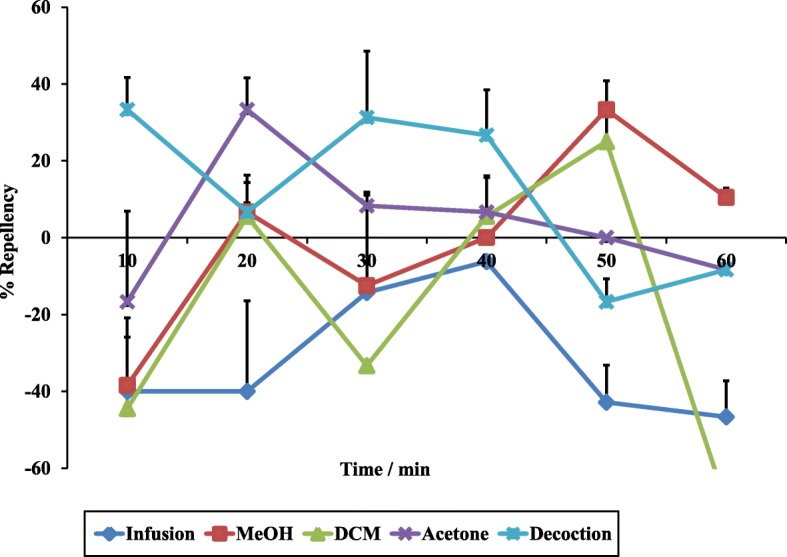
Percentage repellences against *R. appendiculatus* adults for the negative controls (dichloromethane, acetone, methanol, soap-water infusion and the decoction)

The repellent activity of the different negative controls against adults of *R. appendiculatus* was examined over time (Fig. 3). With the exception of one time, the repellency was generally below 5% for the organic extractants. The repellency of the decoction was the highest and diminished over time. The soap-water mixture attracted the ticks rather than repelling them.

### Crude extracts

**Fig. 4:**
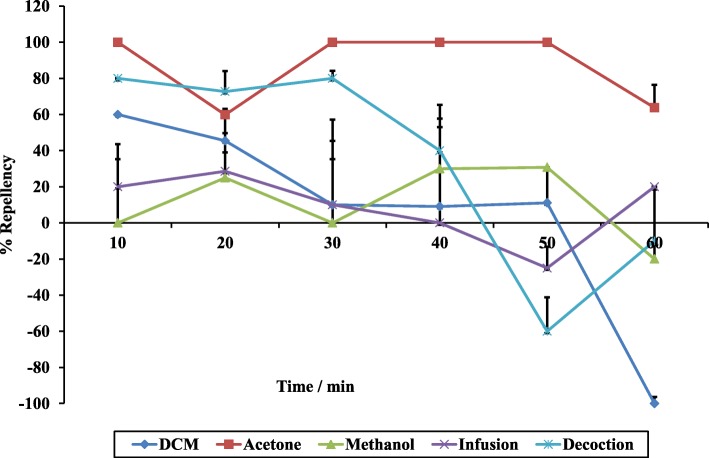
Percentage repellency of 30% crude extracts against *R. appendiculatus* adults over time

The repellency response of *R. appendiculatus* adults to different 30% crude extracts of *R. glabrum* was determined (Fig. 4)*.* It was surprising that the DCM extract attracted 100% of ticks after 1 h. It was also noteworthy that the decoction initially had a good repellency activity against the ticks, but decreased after 50 min, although the difference was not statistically significant (Table 1). On the contrary, the repellency effect of the acetone extract differed significantly from the control treatment with a *p*-value of less than 0.05 (two-tailed test).

**Table 1:** Summary of the repellency effects and statistical analysis of the 30% crude extracts when compared to the negative controls

		Decoction	Infusion	MeOH	Acetone	DCM
Average % repellency		33.7	8.93	11	87.3	5.94
*t*-test	Equal mean variance	0.408	0.0031	0.415	1.5e-5	0.391
	Unequal mean variance	0.419	0.0016	0.416	1.6e-5	0.395
p (2-tail) value		0.298	0.0131	0.69	0.00507	0.174
Mann-Whitney U test		*p* > 0.05	*p* < 0.05	*p* > 0.05	*p* < 0.05	*p* > 0.05

**Fig. 5:**
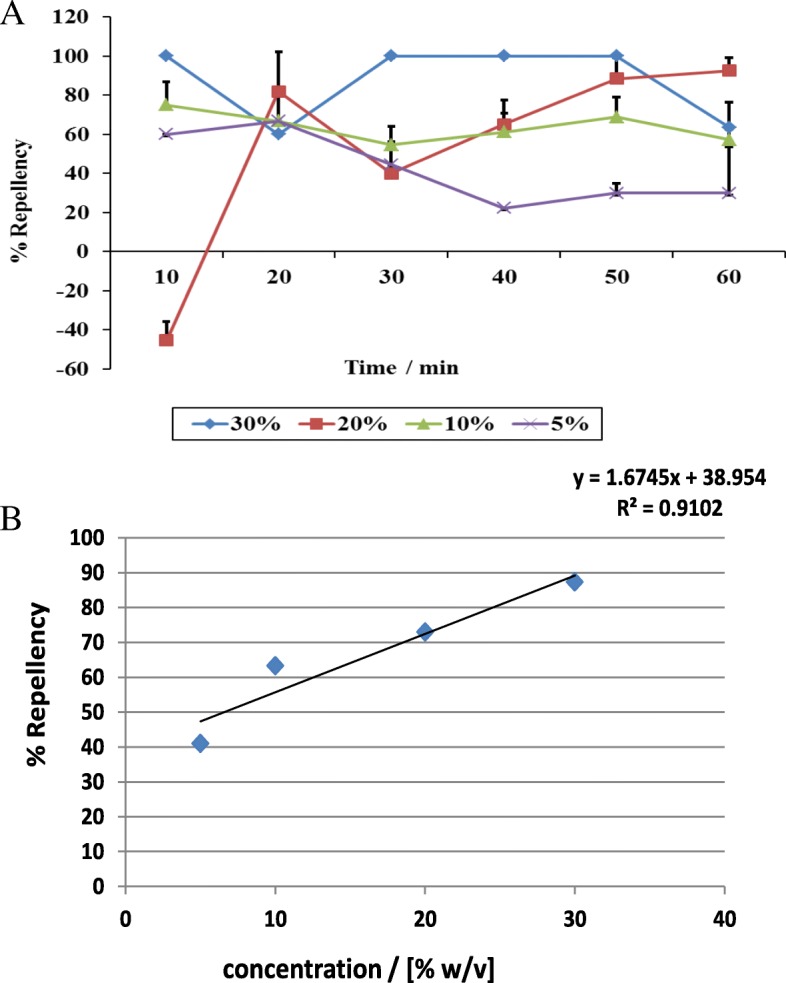
Repellent effects of acetone crude extracts of *R. glabrum* against *R. appendiculatus* adults. **a** Percentage repellency of four concentrations. **b** Relationship between average percentage repellency at different concentrations to determine EC_50_ of 6.6%

The significant repellency of the 30% crude acetone extract led us to evaluate activities at lower concentrations. The 30% acetone extract retained 100% repellency up to the 50-min time point except at the 20 min mark where it dropped briefly to 60% (Fig. 5a). As the concentration decreased to 5%, the extract lost its average repellency activity. The EC_50_ of 6.6% was calculated from the trend line graph of average activities (Fig. 5b).

### Activity of the acetone fractions

**Fig. 6:**
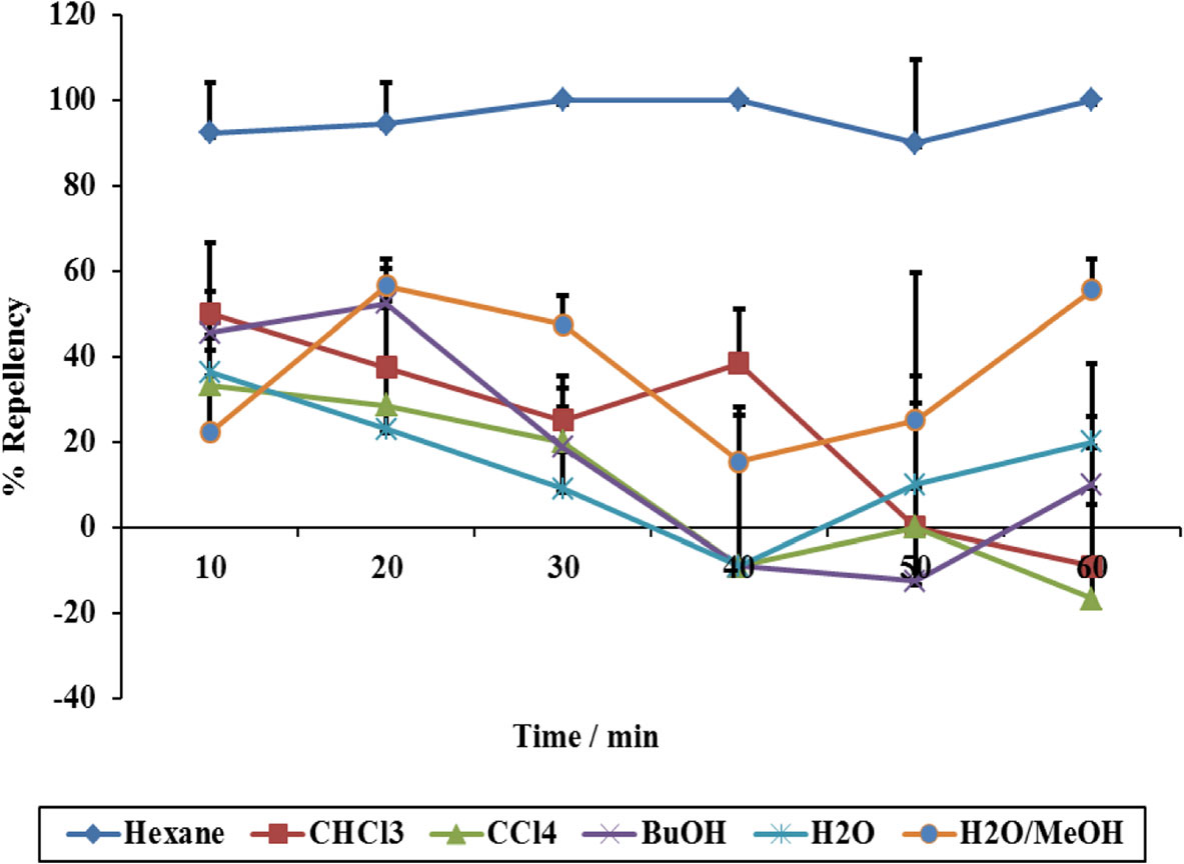
Percentage repellencies of different 30% solvent-solvent fractions against *R. appendiculatus* adults measured over a 60-min time period

Percentage repellencies of the six different fractions were determined (Fig. 6). The hexane fraction had an average repellency of 96.2%, followed by 35% water-in-methanol (36.8%), chloroform (24.0%), butanol (18%), water (14.3%) and, lastly, carbon tetrachloride (9.27%). From these results, it is clear that the bioactive principles are non-polar compounds. The repellency activity value for the hexane fraction was significantly greater than those of the two positive controls, Bayticol (89%) and Amitix (45%) (Fig. 2). although one should remember that different concentrations of hexane fraction were used.

**Fig. 7:**
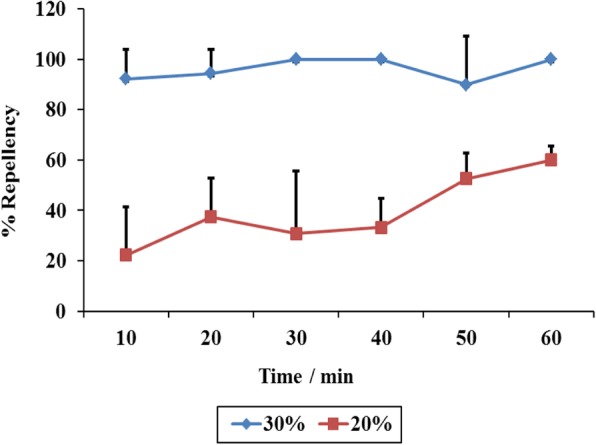
Percentage repellency levels of two hexane fraction concentrations (30 and 20%) against *R. appendiculatus* adults

To determine the efficiency of lower hexane concentrations, the experiment was repeated using 20% hexane. The average repellency drastically dropped to 40% and no lower concentrations were tested (Fig. 7).

### Evaluation of volatility of the bioactive components

**Fig. 8:**
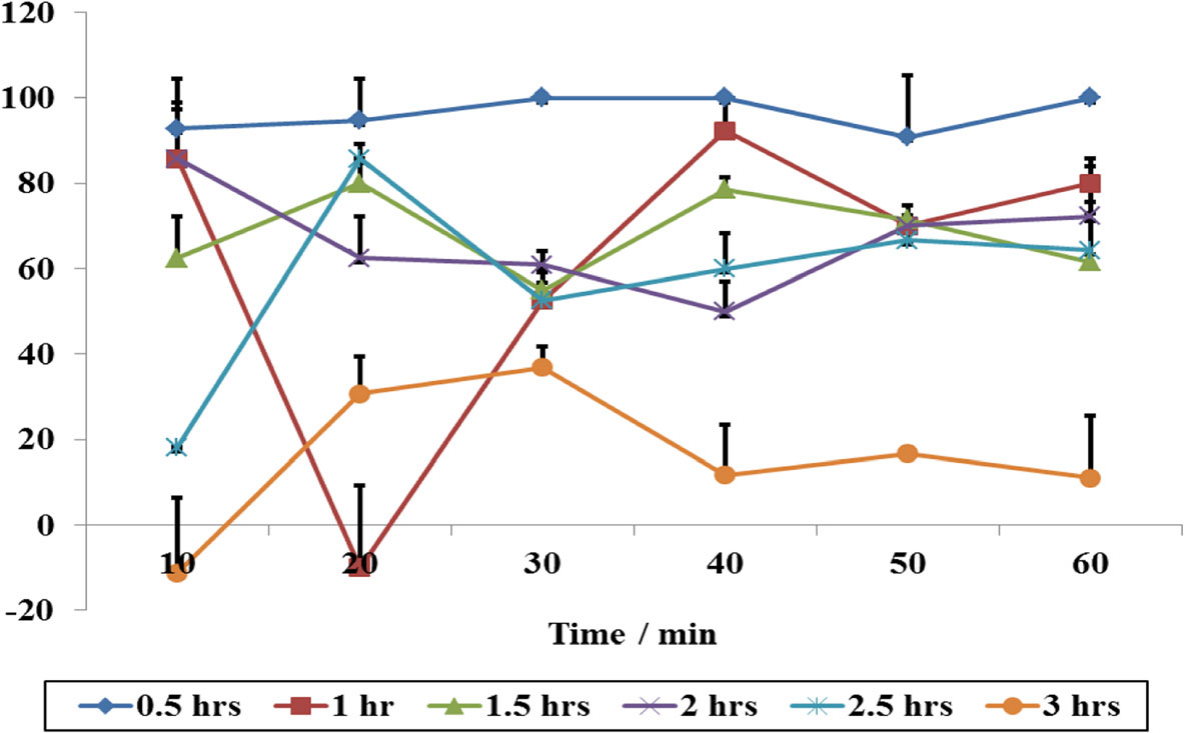
Percentage repellency of 30% hexane fractions against *R. appendiculatus* adults when different drying times of the experimental filter papers were explored

Another aspect considered was longevity of the repellency. Thus, five different drying times (up to 3 h) of the filter paper coated with 30% hexane fractions were explored and the percentage repellencies were determined (Fig. 8). As expected, the average percentage repellencies for 0.5 h drying time gave the highest average activity over the course of the test period of 96.17%. As the drying times increased, the repellency effects dropped to as low as 18% at 3 h, thus indicating that the repellent compounds are volatile. Due to volatility and low concentrations of bioactive components encountered in the present study, *R. glabrum* essential oils may be the best option for effective and practical control of ticks in the field.

### Attempts to isolate the active compounds

Open column vacuum liquid chromatography fractionation on silica gel was conducted in an attempt to isolate the repellent compounds from the bioactive fraction. Solvents of increasing polarity with the non-polar hexane, ethyl acetate, acetone and methanol, were used for further fractionation, resulting in four sub-fractions. The sub-fractions were tested for repellency activity and the ethyl acetate gave the highest repellency activity of 73.6%, followed by acetone (65.9%), hexane (15.0%) and methanol (12.5%). However, when further fractionation of the ethyl acetate sub-fraction was conducted, the resulting fractions lost repellency activities, against *R. appendiculatus* adults.

As bioassay guided fractionation of the acetone extract continued, the hexane subfraction had higher activities (96.2%) than the commercial products. The hexane sub-fraction lost activity while the ethyl acetate sub-fraction retained activity of 73.6%. These activities indicate that the non-polar and intermediate polarity compounds found in the hexane fraction and ethyl acetate fraction had an influence on repellence. By combining the hexane (65.5%), DCM/ethyl acetate (58.7%) and ethyl acetate (57.2%) sub-fractions obtained after conducting further fractionation of the ethyl acetate sub-fraction, the original activity (73.4%) was re-established to prove the existence of synergism between different compounds.

## Discussion

East Coast Fever in cattle results from a strictly trans-stadial transmission of *T. parva* by a three-host tick called *R. appendiculatus* [27]*.* Transmission to the cattle occurs during feeding of the infected *R. appendiculatus* species, whereby *T. parva* sporozoites are released into the feeding site to infect bovine lymphocytes in order to develop into the schizont stage [28]. Thus, cattle become tick reservoirs that transmits parasites to the next feeding vectors once the succeeding infective stage in the life cycle of the parasite is complete. However, the degree of transmission from the infected cattle to the vectors differs between *R. appendiculatus* strains [29]. Therefore, in order to circumvent any possible transmission of the parasites from the infected cattle, the best option is to treat the infected livestock with efficient drugs, while the *T. parva* parasite is undergoing its developmental stages [30].

The leaf extracts of *R. glabrum* have been reported to play a significant role in insect and tick management systems [24, 25]. The reason being that plant-based acaricides provide a low-cost option for effective control of ticks particularly for farmers in the sub-Saharan African countries with poor infrastructure. Therefore, in order to impede transmission of *T. parva* from one vector to another during the feeding cycle of the *R. appendiculatus* ticks, the solution may be to interrupt the feeding process and/or life cycle of the ticks by application of effective repellent extracts prepared from the leaves of *R. glabrum* plants using customised methods*.* Apart from the commercial acaricides, such as amitraz used in a previous study, aqueous extracts of *Lippia javanica* are effective in disturbing the life cycle of the ticks [31]. Other research studies also showed that application of *Tagetes minuta* essential oils delayed moulting of *Hyalomma rufipes* engorged tick nymphs, and also interfered with the egg hatching process of the coleopteran beetle, *Tribolium castaneum* [32, 33]. Hence, the present study opted to investigate the repellent efficiencies of *R. glabrum* leaf components prepared using organic solvent and aqueous extraction techniques against *R. appendiculatus* adult ticks.

The repellency efficiencies of the extracts prepared using the different extraction methods were measured over a 60-min time period at different concentrations using an adaptation of the Climbing Repellency Bioassay method [34]. The decreasing trend up to 30 min observed in the decoction as shown in the results of the repellency activities of the crude extracts, may substantiate the ethnoveterinary use of *R. glabrum*. It is also clear that a 30% acetone extract contained substances that had excellent average repellency activity of 87.3%, compared to the commonly used commercial repellents, Bayticol® (89%) and Amitix® (45%), used at the same concentrations. The effective repellent activity was retained up to a lower concentration of 10%, indicating that the behavioural response of *R. appendiculatus* was dose-dependent as repellent activities of the extract increased with an increase in the concentration and an EC_50_ value of 6.6% (*w*/*v*) was determined. Even though the value was somewhat higher than those reported in the previous studies, with lowest EC_50_ values of 0.45 and 4.7% when extracts were tested against another tick species, *Hyalomma marginatum rufipes* [34, 35], the differences were statistically significant (*P* < 0.05). Furthermore, the decrease in repellency activities of Amitix for the first 30 min followed by an increase is interesting. The decrease may be caused by evaporation of the active volatile compound(s). If these results are confirmed, it may be interesting to track the change in volatile compounds over time using gas chromatography. However, the increase after 30 min was expected to support the perception of the majority of the farmers, veterinarians and para-veterinarians about Amitix as a fast acting amitraz-compound with tick removal or death occurring between 30 min to 3 h [36]. In addition, the efficacy of amitraz using susceptibility tests, spraying and dipping trials on cattle against *Rhipicephalus appendiculatus, Amblyomma variegatum* and *Boophilus decoloratus* tick species was also assessed [37]. The results after spraying confirmed this unique phenomenon whereby ticks started dislodging from the animals between 30 min and 1 h.

Furthermore, the present study showed high repellence activities of Bayticol against *R. appendiculatus* adults. A previous study also reported a significant repellent effect of Bayticol when applied on hairs clipped from cattle and sheep against *Ixodes ricinus*, *Dermacentor reticulatus* and *Rhipicephalus sanguineus* species [38]. Thus, when mixed with Bayticol-treated hair, the ticks tried to escape and did not seek shelter inside the hair. Bayticol showed toxicity effects against *I. ricinus* and *D. reticulatus.* The ticks died within 5–12 h of coming into contact with the cattle hair of animals treated 3 weeks previously and within 6–9 h after contact with treated sheep hair. Therefore, it is clear that Bayticol possesses both toxicant and repellent properties against various tick species.

After bioassay-guided fractionation of the acetone extract, the hexane fraction showed higher activities (96.2%) than the commercial products. The hexane sub-fraction lost activity while the ethyl acetate sub-fraction retained activity of 73.6%. These activities indicate that the non-polar and polar compounds found in the hexane fraction influenced each other to bring about the higher repellence activity. Thus, this is one of the few cases where unambiguous proof of synergism between different compounds was found since activity was re-established after combining the sub-fractions. It is highly recommended that future studies should isolate and characterise the repelling agents in order to be certain of their nature and safety as the resulting botanicals may eventually be applied topically on livestock, handled by man and exposed to the environment.

Moreover, a previous investigation compared the infection prevalence between adult and nymphal ticks feeding on *T. parva* infected cattle [39]. The results showed 5–20 times higher prevalence of infection in adult ticks. Therefore, it would be informative in future to assess nymphal repellency of the 30% acetone extract since slightly lower prevalence of infection was observed, as well as variations in the repellency for male and female adult ticks. It will also be interesting to assess repellency effects of the acetone extract against different isolates of *R. appendulatus* ticks displaying different mitochondrial genotypes.

## Conclusion

The idea behind using a soap water mixture was to see if this mixture would extract non-polar compounds from the leaves. In this assay, the treatment of the extract after extraction may have led to the evaporation of the volatile compounds that are responsible for the repellent activity. Previous work could prove that in in vivo experiments, water supplemented with 1% of a surfactant and vegetable oil extract of *Maurea edulis* tuber was as effective as Amitraz in protecting cattle against natural infections by ticks [40]. In this case, the activity may not have been repelling, but killing the ticks because the effect lasted more than 7 weeks. It may be worthwhile to focus on killing rather than repelling the ticks and the possibility of extracting non-polar compounds with a soap-water-oil mixture should be considered more widely as this could be a way of effectively protecting animals against ticks in poor rural communities. Due to the volatility of the bioactive compounds these plant extracts may not be that useful in the veld, but could be useful in animals kept in a kraal.

## Methods

### Plant drying and processing

Fresh leaves of *Rotheca glabrum* (Verbenaceae) were collected from Vhembe region (22^o^56’ S 30^o^28’ E) of the Limpopo Province, South Africa. The leaves were washed using tap water, dried at room temperature in the Ethno-Veterinary medicine (EVM) laboratory at the Agricultural Research Council/Onderstepoort Veterinary Institute (ARC/OVI) (25^o^ 39.071′ S 28^o^ 11.033′ E) in Gauteng Province, South Africa. The dry leaves were ground into powder using a Büchi Grinder B-400 (Labotec). The dry powder was then divided into brown closed bottles to avoid oxidation and stored in the laboratory at room temperature.

### Cold extraction

The dry powder prepared from the leaves of *R. glabrum* were extracted using either dichloromethane (non-polar), acetone (intermediate polar) or methanol (polar) [41]. In brief, 3 grams of the dry powder was weighed separately into three solvent-labelled 50 mL centrifuge tubes. Thirty mL of the solvent was added into the centrifuge tube corresponding to its label. The mixtures were shaken vigorously for 15 min using a benchtop shaker (Labotec, model no: 202). Two ml of the extracts were removed from the upper liquid phase of each tube and placed separately into 40 mL glass beakers. These were then dried and weighed to determine the concentration of the extracts.

The remaining 28 mL solutions were centrifuged for 5 min at 3500 rpm, filtered through Whatman no. 1 filter papers and the supernatants were collected into three labelled 50 mL glass beakers. The process was repeated and the supernatants collected from the second extraction were combined with the first collected supernatants to complete extraction and stored in a refrigerator at 4 °C to avoid fungal growth [42].

### Preparation of the decoction

Five grams of the dry powder and 30 mL of water were placed in a 100 ml glass beaker and boiled for 10 min at 80-100 °C. The mixture was allowed to cool at room temperature and then filtered through a Whatman no. 1 filter paper into a clean 250 ml conical flask as previously adopted [43]. The prepared extract was then stored in a fridge at 4 °C to avoid fungal growth.

### Preparation of soap-water infusion

Five grams of the dry powder was weighed into a 100 mL glass beaker and 0.2 g of Surf washing powder (a widely used commercial detergent freely available in rural communities containing biodegradable silicates, soda ash and phosphates) was added to 30 mL of warm water (55-65 °C) to act as a water softener. The mixture was left to stand for 30 min on the bench. The solution was then strained through a clean dry cloth into a 250 mL conical flask. Paraffin (1.2 mL) and 0.15 g of sodium bicarbonate of soda were added and the solution incubated for 2 weeks in an oven at 30 °C [43]. The prepared extract was then stored in a refrigerator at 4 °C to avoid fungal growth. The rationale for adding paraffin and sodium bicarbonate to the extract and then incubation after removal of the plant material is not clear. This may have been added to protect the extract from fungal attack. It could also have been developed for in vivo work with the paraffin acting as a sticking agent on the animals. To yield comparable results, we followed the method as published.

### Rearing of developmental stages of ticks

Adult stages of *R. appendiculatus* ticks used in this study were obtained from laboratory colonies at Clinvet Ltd. (South Africa) and were maintained on rabbits in the Department of Biology at the University of Limpopo (Medunsa Campus). The tick naïve rabbits used in this study, were bred and maintained at the Animal Production Unit of the Department of Biology, University of Limpopo (Medunsa Campus). In the laboratory, the *R. appendiculatus* ticks were maintained at 25 ± 1 °C, 75 ± 5 °C relative humidity (RH) and natural day/night regimen. Ethical clearance for the study, was granted by the Medunsa Animal Ethics Committee. Used rabbits were euthanized by a qualified veterinarian, who administered a sedative drug prior to a euthanizing drug. Rabbit carcasses together with used ticks were incinerated at the Medunsa Campus, University of Limpopo incinerator.

### Climbing repellency bioassay and experimental procedure

#### Climbing repellency bioassay

A climbing repellency bioassay was adopted as previously described [34] to test for the repellent properties of *R. glabrum*. In brief, an 80 mL glass beaker filled with polystyrene was firmly inserted in the centre of a 250 mL glass beaker. The polystyrene provided support to the vertically inserted glass rod (length 22.1 cm) and also served as a platform on which ticks were placed. Additional polystyrene was used to support the 80 mL glass beaker, preventing it from falling in the 250 mL glass beaker. Water was poured into the 250 mL glass beaker to completely surround the 80 mL glass beaker and to height just below its rim. This was done to discourage ticks from crawling away from the polystyrene platform and to stabilise the humidity. The top 5 cm of the glass rod was covered with Whatman no. 1 filter paper (2.5 × 5 cm) to which a 30% (*w*/*v*) concentration of the plant extract and 30% (w/v) of positive controls (Amitix and Bayticol) was added. A second piece of filter paper of the same type and size was fixed just below the top one to serve as a neutral zone. No extract was added to the neutral zone filter paper as it provided an alternative questing place for the ticks, comparable with the treatment filter paper. Similarly, a negative control was set up with the appropriate solvent only on the top filter paper. The solvent in both the treatment and control filter papers was allowed to evaporate for 15 min before the start of the experiment.

### Experimental procedure

Ten unsexed *R. appendiculatus* adults were placed on a platform of the treatment apparatus and subsequently the same was done on the control apparatus. Prior to the start of the experiment the ticks were allowed a 15 min acclimatisation period, following which their position on the glass rod was noted at 10 min intervals up to 60 min. After each 10 min interval ticks on the glass rods were moved back to the polystyrene platform.

Ticks on the extract filter paper were considered not to be repelled by the extract and ticks found mainly on the neutral filter paper or glass rod below the extract were considered to be repelled. Similarly, ticks found on the negative solvent control were considered not repelled and ticks found on the neutral filter paper or glass rod below the negative solvent control paper were considered to be repelled by the solvent. The same argument held for the positive controls that were used. Ticks which fell into the surrounding water were dried using Kimberly-Clark paper towel and carefully replaced onto the platform using forceps.

Different concentrations of the extracts (in the original solvent) and positive controls (Amitix and Bayticol dissolved in water), from 30% down to 5% based on activity found, were used. Three replications were performed for each extract concentration.

### Data analysis

The number of ticks and their position on the glass rod were recorded. Ticks that returned or quested below the treatment filter papers were regarded to have been repelled. The percentage repellency was calculated using the following adopted formula [44].

$$ \%\mathrm{Repellency}=100\hbox{-} \frac{\left[\mathrm{mean}\ \mathrm{number}\ \mathrm{of}\ \mathrm{ticks}\ \mathrm{on}\ \mathrm{test}\right]}{\left[\mathrm{mean}\ \mathrm{number}\ \mathrm{of}\ \mathrm{ticks}\ \mathrm{on}\ \mathrm{control}\right]}\times 100 $$

The average percentage repellency was calculated per an hour. Lower concentrations of the bioactive extract were made, used to test for repellent activities and to determine the effective concentration able to repel 50% of the ticks.

A non-parametric Mann-Whitney U test (Stat Cat v. 3.7.1) was selected as the main statistical analysis test on the grounds that the data obtained were quantitatively discreet and distribution-free [45].

### Phytochemistry

**Fig. 9:**
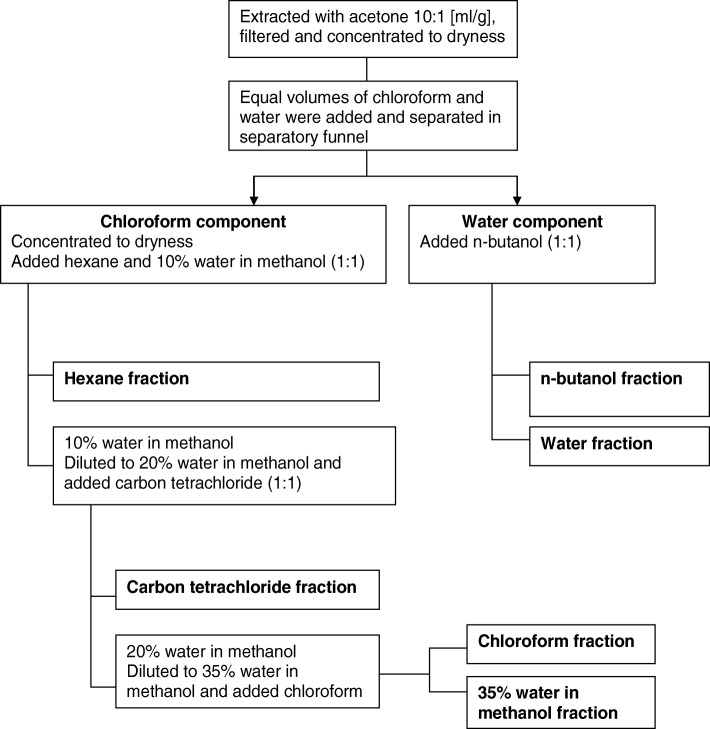
Flow diagram showing extract solvent-solvent fractionation process [46]

Based on the repellent activity shown against the ticks, the acetone extract of *R. glabrum* was selected and subjected to a solvent-solvent fractionation in a first step towards isolating the active compound. The separation was undertaken with immiscible solvents to fractionate components with different polarities. The procedure used for the group separation process was based on a procedure employed by the National Cancer Laboratory in the USA and was modified slightly as illustrated in Fig. 9 [46]. The repellent activities of all fractions were determined.

The volatility of the bioactive fractions was determined after a period of 3 h. In an attempt to isolate the active compound(s), vacuum liquid chromatography (VLC) on Silica Gel open column chromatography using four organic solvents, namely; hexane, ethyl acetate, acetone and methanol was applied to sub-fractionate the bioactive components. The repellence activity of the individual fractions and some combinations thereof were determined.

## Abbreviations

%: Percentage
ARC: Agricultural Research Council
BuOH: n-butanol
CCl_4_: Carbon tetrachloride
CHCl_3_: Chloroform
cm: Centimetre
*D. reticulatus*: Dermacentor reticulatus
DCM: Dichloromethane
EC_50_: Effective concentration of the extract to repel 50% of the ticks
ECF: East Coast Fever
EVM: Ethno-veterinary medicine
g: Gram
H_2_O: Water
H_2_O/MeOH: Water in methanol
hr: Hour
hrs: Hours
I. ricinus: Ixodes ricinus
MeOH: Methanol
min: Minute
mL: Milliliter
NRF: National Research Foundation
^o^C: Degrees Celsius
OVI: Onderstepoort Veterinary Institute
p: Probability
*R. appendiculatus*: Rhipicephalus appendiculatus
*R. glabrum*: Rotheca glabrum
RH: Relative humidity
rpm: Revolutions per minute
SACNASP: South African Council for Natural Scientific Professions
SAVC: South African Veterinary Council
*T. parva*: *Theileria parva*
*T. taurotragi*: *Theileria taurotragi*
TOXSA: Toxicology Society of South Africa
US$: United States dollar
VLC: Vacuum liquid chromatography
*w*/*v*: Weight per volume

## References

[1] Parola PRD (2001). Ticks and tick-borne bacterial diseases in humans: an emerging infectious threat. Clin Infect Dis.

[2] Brites-Neto J, Duarte KMR, Martins TF (2015). Tick-borne infections in human and animal population worldwide. Vet World.

[3] Guglielmone AA, Robbins RG, Apanaskevich DA, Petney TN, Estrada-Penaz G, Horak IG (2010). The Argasidae, Ixodidae and Nuttalliellidae (Acari: Ixodida) of the world: a list of valid species names. Zootaxa.

[4] Carrol JF, Solberg VB, Klun JA, Kramer M, Debboun M (2004). Comparative activity of Deet and AI3 – 37220 repellents against the ticks *Ixodes ascapularis* and *Amblyomma americanum* (Acari : Ixodidae) in laboratory bioassays. J Med Entomol.

[5] Lane RP, Crosskey RW, Lane RP, Crosskey RW (1996). House -fly, blow-flies, and their allies (Calyptrate Diptera). Medical insects and arachnids.

[6] Liyanaarachchi DR, Jinadasa HRN, Dilrukshi PRMP, Rajapakse RPVJ (2013). Epidemiological study on ticks in farm animals in selected areas of Sri Lanka. Trop Agric Res.

[7] Lwande W, Ndakala AJ, Hassanali A, Moreka L, Nyandat M, Ndungu M (1999). *Gynandropsis gynandra* essential oil and its constituents as tick (*Rhipicephalus appendiculatus*) repellents. Phytochemistry.

[8] Walker AR, Bouattour JL, Camicas A, Estrada-Pena A, Horak IG, Latiff AA (2003). Ticks of domestic animals in Africa: a guide to identification of species, bioscience report.

[9] Fletcher WA (1984). A guide to practical tick control in southern Africa.

[10] Mbogo SK, Kariuki DP (1995). Immunisation against East Coast fever: possible ways of reducing costs. E Afr agric For J.

[11] Walker JB, Keirans JE, Horak IG (2005). The genus *Rhipicephalus (*Acari, Ixodidae*):* a guide to the brown ticks of the world.

[12] de Castro JJ (1997). Sustainable tick and tick-borne disease control in livestock improvement in developing countries. Vet Parasitol.

[13] Mukhebi AW, AW PBD, Kruska R (1992). Estimated economics of theileriosis control in Africa. Prev Vet Med.

[14] Kivaria FM (2006). Estimated direct economic costs associated with tick-borne diseases on cattle in Tanzania. Trop Anim Health Prod.

[15] Eisler MC, Torr SJ, Coleman PG, Machila N, Morton JF (2003). Integrated control of vector-borne diseases of livestock – pyrethroids: panacea poison?. Trends Parasitol.

[16] Artigas-Jerónimo S, De La Fuente J, Villar M (2018). Interactomics and tick vaccine development: new directions for the control of tick-borne diseases. Expert Rev Proteomics.

[17] Radley DE, Brown CGD, Burridge MJ, Cunningham MP, Kirimi IM, Purnell RE, Young AS (1975). East Coast fever: 1. Chemoprophylactic immunization of cattle against *Theileria parva* (Muguga) and five theilerial strains. Vet Parasitol.

[18] Uilenberg G, Silayo RS, Mpangala C, Tondeur W, Tatchell RJ, Sanga HJ (1977). Studies on Theileriidae (Sporozoa) in Tanzania. X. A large-scale field trial on immunization against cattle theileriosis. Tropenmed Parasitol.

[19] Morzaria S, Nene V, Bishop R, Musoke A (2000). Vaccines against *Theileria parva*. Ann N Y Acad Sci.

[20] Sitt T, Poole EJ, Ndambuki G, Mwaura S, Njoroge T, Omondi GP (2015). Exposure of vaccinated and naive cattle to natural challenge from buffalo-derived *Theileria parva*. Int J Parasitol Parasites Wildl.

[21] Nene V, Kiara H, Lacasta A, Pelle R, Svitek N, Steinaa L (2016). The biology of *Theileria parva* and control of East Coast fever - current status and future trends. Ticks Tick Borne Dis.

[22] Graf JF, Gogolewski R, Leach-Bing N, Sabatini GA, Molento MB, Bordin EL (2004). Tick control: an indus-try point of view. Parasitology.

[23] Ghosh S, Azhahianambi P, Yadav MP (2007). Upcoming and future strategies of tick control: a review. J Vector Borne Dis.

[24] Mabogo DEN. The ethnobotany of the Vhavenda, MSc thesis: University of Pretoria; 1990. www.up.ac.za.

[25] Hutchings A, Scott AH, Lewis G, Cunningham A (1996). Zulu medicinal plants: an inventory.

[26] Roberts M (1997). Indigenous healing plants.

[27] Norval RAI, Perry BD, Young AS, Lawrence JA, Mukhebi AW, Bishop R (1992). The epidemiology of theileriosis in Africa.

[28] Olds CL, Mason KL, Scoles GA (2018). *Rhipicephalus appendiculatus* ticks transmit *Theileria parva* from persistently infected cattle in the absence of detectable parasitemia: implications for East Coast fever epidemiology. Parasit Vectors.

[29] Young AS, Dolan TT, Mwakima FN, Ochanda H, Mwaura SN, Njihia GM (1995). Estimation of heritability of susceptibility to infection with *Theileria parva* in the tick *Rhipicephalus appendiculatus*. Parasitology.

[30] Schorderet-Weber S, Noack S, Selzer PM, Kaminsky R (2017). Blocking transmission of vector-borne diseases. Int J Parasitol Drugs Drug Resist.

[31] Madzimure J, Nyahangare ET, Hamudikuwanda H (2011). Acaricidal efficacy against cattle ticks and acute oral toxicity of Lippia javanica (Burm F.) Spreng. Trop Anim Health Prod.

[32] Krishna A, Prajapati V, Bhasney S, Tripathi AK, Kumar S (2005). Potential toxicity of new genotypes of *Tagetes* (Asteraceae) species against stored grain insect pests. Int J Trop Insect Sci.

[33] Nchu F, Magano SR, Eloff JN (2012). *In vitro* anti-tick properties of the essential oil of *Tagetes minuta* L. (Asteraceae) on *Hyalomma rufipes* (Acari: Ixodidae). Onderstepoort J Vet Res.

[34] Mkolo MN, Magano SR (2007). Repellent effects of the essential oil of *Lavendula angustifolia* against adults of *Hyalomma marginatum rufipes*. J S Afr Vet Assoc.

[35] Nchu F, Magano SR, Eloff JN (2016). Repellent activities of dichloromethane extract of *Allium sativum* (garlic) (Liliaceae) against *Hyalomma rufipes* (Acari). J S Afr Vet Assoc.

[36] Bardosh K, Waiswa C, Welburn SC (2013). Conflict of interest: use of pyrethroids and amidines against tsetse and ticks in zoonotic sleeping sickness endemic areas of Uganda. Parasit Vectors.

[37] Kagaruki LK (1996). The efficacy of amitraz against cattle ticks in Tanzania. Onderstepoort J Vet Res.

[38] Mehlhorn H, Schumacher B, Jatzlau A, Abdel-Ghaffar F, Al-Rasheid KA, Bhushan C (2012). The effects of flumethrin (Bayticol® pour-on) on European ticks exposed to treated hairs of cattle and sheep. Parasitol Res.

[39] Ochanda H, Young AS, Wells C, Medley GF, Perry BD (1996). Comparison of the transmission of *Theileria parva* between different instars of *Rhipicephalus appendiculatus*. Parasitology.

[40] Nyahangare ET, Mvumi MV, Magona C, Eloff JN (2017). *In vivo* efficacy of *Aloe vera*, *Cissus quadrangularis* and *Maerua edulis* aqueous extracts against cattle ticks. Ind Crop Prod.

[41] Eloff JN (1998). Which extractant should be used for the screening and isolation of antimicrobial components from plants?. J Ethnopharmacol.

[42] Eloff JN, McGaw LJ, Ahmad I, Aqil F, Owais M (2006). Plant extracts used to manage bacterial, fungal, and parasitic infections in southern Africa. Modern Phytomedicine: turning medicinal plants into drugs.

[43] Berger A, Mugoya C (1995). Natural plant products as pesticides. Proceedings from the first national symposium in Zambia, report 4.

[44] Ibrahim J, Zaki Z (1998). Development of environment-friendly insect repellents from the leaf oils of selected Malaysian plants. ARBEC.

[45] Bland M (1987). An introduction to medical statistics.

[46] Eloff JN (1998). The presence of antibacterial compounds in *Anthocleista grandiflora* (Loganiaceae). S Afr J Bot.

